# Different Involvement of ASIC and TRPA1 in Facial and Hindpaw Allodynia in Nitroglycerin-Induced Peripheral Hypersensitivities in Mice

**DOI:** 10.3390/life12091294

**Published:** 2022-08-23

**Authors:** Sol-Ji Kim, Ji-Hee Yeo, Seo-Yeon Yoon, Dae-Hyun Roh

**Affiliations:** 1 Department of Oral Physiology, School of Dentistry, Kyung Hee University, Seoul 02447, Korea; 2 Department of Pet Total Care, Division of Business and Fine Art, Daejeon Health Institute of Technology, Daejeon 34504, Korea

**Keywords:** migraine, nitroglycerine, hypersensitivity, acid-sensing ion channel, transient receptor potential ankyrin type 1

## Abstract

The pathophysiological mechanism underlying migraine-associated peripheral hypersensitivity remains unclear. Acid-sensing ion channels (ASICs) and transient receptor potential ankyrin 1 (TRPA1) are known to be causative pathogenic factors of mechanical and cold allodynia, respectively. Here, we sought to investigate their involvement in cold and mechanical allodynia of the face and hindpaws, respectively, in a mouse model of repetitive nitroglycerin (NTG)-induced migraine. NTG (10 mg/kg) was administered to the mice every other day for 9 days, followed 90 min later by HC-030031 (a TRPA1 blocker) or amiloride (a non-selective ASIC blocker). Mechanical or cold sensitivity of the hindpaw and facial regions was quantified using von-Frey filaments or acetone solution, respectively. Immunohistochemistry revealed that c-Fos expression was significantly increased in the trigeminal nucleus caudalis region but not in the spinal cord. Amiloride treatment only reduced NTG-induced hindpaw mechanical allodynia, whereas HC-030031 treatment only improved facial cold allodynia. Interestingly, the number of c-Fos positive cells decreased to a similar level in each drug treatment group. These findings demonstrate that facial cold allodynia and hindpaw mechanical allodynia are differentially mediated by activation of TRPA1 and ASIC, respectively, in mice with repetitive NTG-induced hypersensitivity.

## 1. Introduction

In general, migraine is a severe headache disorder caused by various factors and characterized by unilateral and pulsatile pain [1]. As the headache progresses, the patient may become sensitive to various external stimuli that are normally harmless, such as ambient light or sound, and may exhibit hypersensitivity to mechanical, cold, or thermal stimuli in the head, neck, and non-cephalic regions [2]. Although central sensitization may contribute to peripheral hypersensitivity, the exact mechanism involved is not well known [2,3].

Nitroglycerin (NTG), a nitric oxide donor and effective vasodilator, is commonly used to treat patients with angina pectoris. The main side effects of NTG include indigestion, erythema, muscle pain, and migraine [4]. Migraine can develop within a few minutes or within 4 h after NTG administration, and the systemic administration of NTG has been reported to produce a nociceptive response in mice and rats [5]. Experimentally, a single injection of NTG in mice was shown to induce acute mechanical and cold hypersensitivity, lasting up to 4 h after injection [3,6]. Several recent papers demonstrated chronic hypersensitivity to mechanical stimuli in mice following repeated systemic administration of NTG, which mimicked the chronic migraine headache observed in patients [7,8,9]. Therefore, since mechanical and cold hyperalgesia are indicators of migraine-related peripheral hypersensitivities, a mouse animal model of NTG-derived peripheral hyperalgesia is considered useful for studying the pathogenesis of migraine-related sensory abnormality [7,10]. We previously reported that pain to cold stimulation increased in the face region, whereas allodynia to mechanical stimulation was significantly induced in only the hindlimb region of the mouse model [11]. Moreover, we verified that NTG-induced heat hypersensitivity is dependent on capsaicin-sensitive primary afferents, while mechanical hypersensitivity in the hindpaw and cold allodynia in the facial region are not.

A recent study found that nerve fibers distributed in the dura mater express the acid-sensing ion channel (ASIC), and these receptors contribute to the development of peripheral hypersensitivity by recognizing the decrease in pH due to dural inflammation and ischemic factors during migraine attacks [12]. In addition, Yan et al. reported that the ASIC3 subtype is important for the excitation of dura mater nerve fibers and is mainly activated by mast cell-derived substances in inflammatory conditions [13]. Recently, the use of transient receptor potential type V1 (TRPV1) receptor antagonists has been explored as a treatment for migraine [14,15], and increased c-Fos protein expression in the trigeminal nucleus caudalis (TNC) was confirmed in experimental animal models [16]. Although TRPV1 primarily responds to thermal hyperalgesia, transient receptor potential ankyrin type 1 (TRPA1) and transient receptor potential type M8 (TRPM8) have been identified as receptors for cold stimuli and are being applied to research on cold hyperalgesia and allodynia [17]. Demartini et al. confirmed the effect of ADM12, a TRPA1 antagonist, through a facial formalin pain test in an NTG-derived migraine-induced rat model [18]. However, it is unclear whether there is a direct relationship between ASIC and TRPA1 in the development of facial cold allodynia and hindlimb mechanical allodynia in an animal model of chronic migraine induced by repeated NTG injection.

Therefore, in this study, we utilized the NTG-derived chronic migraine mouse model established in previous studies to verify the effects of ASIC or TRPA1 receptor antagonist treatment on mechanical and cold allodynia occurring in the facial region and hind limbs, respectively. In addition, we investigated whether significant changes in c-Fos protein expression in NTG-injected animals were observed in the TNC and the lumbar spinal dorsal horn and examined the effects after treatment with the ASIC or TRPA1 receptor antagonist.

## 2. Materials and Methods

### 2.1. Animals

Male C57BL/6NTac mice (25–30 g) were purchased from DBL Animal Inc. (Seoul, Korea). In total, 27 mice were used for pain behavior testing and 20 mice for immunohistochemistry. Mice were habituated to colony cages with free access to water and pelleted feed. They were housed in a standard animal facility maintained on a 12-h light/dark cycle (lights on at 07:00 am) with a constant room temperature (23 ± 2 °C). Mice were acclimated for at least one week prior to the experiment. The protocols for this study were approved by the Kyung Hee University Institutional Animal Care and Use Committee (KHUASP[SE]-16-058). All experimental procedures using animals were performed in accordance with National Institutes of Health guidelines (NIH publication No. 86-23, revised 1985).

### 2.2. Drug Administration

Repetitive injection of NTG was used to produce facial and peripheral hypersensitivity in this mouse model of chronic migraine. Before intraperitoneal injection, the stock solution of NTG (5.0 mg/mL NTG in 30% alcohol, 30% propylene glycol, and water) was dissolved in 0.9% saline to a concentration of 10 mg/kg. NTG was repetitively administrated every other day for 9 days. “Pre-injection basal pain responses” were measured prior to every NTG injection on days 1–9, while the responses observed from days 11 to 21 were considered the “threshold response” (pre-injection basal response). Post-treatment measurement responses were obtained 2 h after each NTG injection (post-treatment response). All experiments were performed with researchers blinded to the pharmacological treatment of the mice [11,19].

After NTG treatment, amiloride, an ASICs blocker (Sigma-Aldrich, Merck Korea, Seoul, Korea) or HC-030031, a selective TRPA1 blocker (Tocris, Bio-Techne Korea, Gyeonggi-do, Korea) were used as experimental anti-allodynic drugs. Amiloride was diluted in 0.9% saline containing 40% dimethyl sulfoxide (DMSO) at a dose of 10 mg/kg, and HCG30031 was dissolved in 0.9% saline containing 10% DMSO at a concentration of 100 mg/kg. Each drug was administered 90 min after NTG treatment, and the pain response was evaluated 30 min later.

### 2.3. Mechanical and Cold Allodynia Tests

Paw withdrawal and facial pain responses were used to quantify mechanical sensitivity. Mice were first acclimated for 30 min to an acrylic cylinder (6.5 cm in diameter and 17 cm in height) on a metal mesh grid prior to testing for mechanical allodynia. To evaluate mechanical allodynia, an ascending series (0.008, 0.02, 0.04, 0.07, 0.16, 0.4, and 0.6 g) of von-Frey filaments (North Coast Medical, Morgan Hill, CA, USA) were applied to the mid-plantar region of the hindpaw and to the whisker pad. Each monofilament, starting with the lowest force (0.008 g), was delivered six times before the next higher force monofilament was tested. Once consistent withdrawal was noted (i.e., the filament that evoked a response in three out of the six trials was defined as the 50% mechanical withdrawal threshold), the test was considered complete [11].

To test cold allodynia, a 0.02 mL acetone solution was applied on both hindpaws, or on the whisker pad, with a 1 cc syringe connected to PE-10 tubing [11,20]. The duration (sec) of withdrawal response (e.g., lifting, shaking, or licking the hind paws and grooming behavior) was counted.

### 2.4. Immunohistochemistry

To investigate the change in c-Fos protein expression in the TNC and lumbar spinal cord regions of NTG-treated animals by drug, another set of mice was sacrificed 2 h after the last injection of NTG (9 days after the first NTG injection). The spinal c-Fos expression increases if the hind-paw pain response is directly correlated with the systemic effects of NTG. Alternatively, it may be associated with NTG-induced TNC stimulation, which causes increased c-Fos expression in TNC. Animals were deeply anesthetized with 5% isoflurane and perfused transcardially with 0.1 mol/L phosphate buffer saline (PBS, pH 7.4), followed by a fixative containing 4% paraformaldehyde in PBS (50 mL). The TNC and spinal cord were removed immediately after perfusion, postfixed in the same fixative for 4 h, and then cryoprotected in 30% sucrose in PBS for 48 h (pH 7.4). Thirty-micrometer-thick transverse frozen sections were cut through the TNC and spinal cord using a cryostat (Leica Microsystems, Wetzlar, Germany). After elimination of endogenous peroxidase activity with 3% hydrogen peroxide in PBS and preblocking with 3% normal goat serum and 0.3% Triton X-100 in PBS for 1 h at room temperature, the sections were incubated in polyclonal rabbit anti-c-Fos antibody (1:1000, Santa Cruz Biotechnology Inc., Dallas, TX, USA) overnight at 4 °C.

After several PBS washes, both the TNC and lumbar spinal cord tissue sections were incubated with a secondary biotinylated anti-rabbit antibody (1:200, Vector Laboratories, Burlingame, CA, USA) for 1 h at room temperature and then processed using the avidin-biotin method (Elite ABC; Vector Laboratories, Newark, CA, USA). Fos-immunoreactive (ir) cells were visualized using a 3-3-diaminobenzidine reaction intensified with 0.2% nickel chloride.

### 2.5. Image Analysis

The TNC and lumbar spinal dorsal horn tissue sections were scanned using the brightfield and fluorescent microscope ECLIPSE 80i (Nikon Corp., Kanagawa, Japan) and digitized using a cooled CCD camera (Cool Snap ES model, Nihon Roper, Tokyo, Japan). Six nonadjacent tissue sections of TNC or lumbar spinal cord per mouse were randomly selected and quantitatively analyzed using a computer-assisted image analysis system (MetaMorph version 7.7.2.0, Westchester, PA, USA). The shape factor was set to a range of 0.5 to 1.0, and c-Fos-ir cells were counted only if they were at least 30% darker than the average gray level of each image [21]. The expression of c-Fos in the lumbar spinal dorsal horn was quantified in the following three spinal cord dorsal horn regions: (1) the superficial dorsal horn (SDH, laminae I and II); (2) the nucleus proprius (NP, laminae III and IV); and (3) the neck region (NECK, laminae V and VI). These regions were identified based on cytoarchitectonic criteria as defined by Abbadie and Besson [22,23]. The average number of c-Fos-ir cells was obtained per section from each animal. These values were averaged across each group, and all analytical procedures described above were blindly performed without advance knowledge of the experimental conditions.

### 2.6. Statistical Analysis

All values are expressed as the mean ± standard error of the mean. A two-way repeated-measures analysis of variance followed by a posthoc Bonferroni test was conducted in the mechanical and cold allodynia tests. In addition, the unpaired *t*-test was used to determine differences in the number of c-Fos-ir cells in the TNC and lumbar spinal cord regions. All statistical analyses were performed using GraphPad Prism (Version 6.0, GraphPad Software, San Diego, CA, USA), and all *p*-values of < 0.05 were considered statistically significant.

## 3. Results

**Figure 1:**
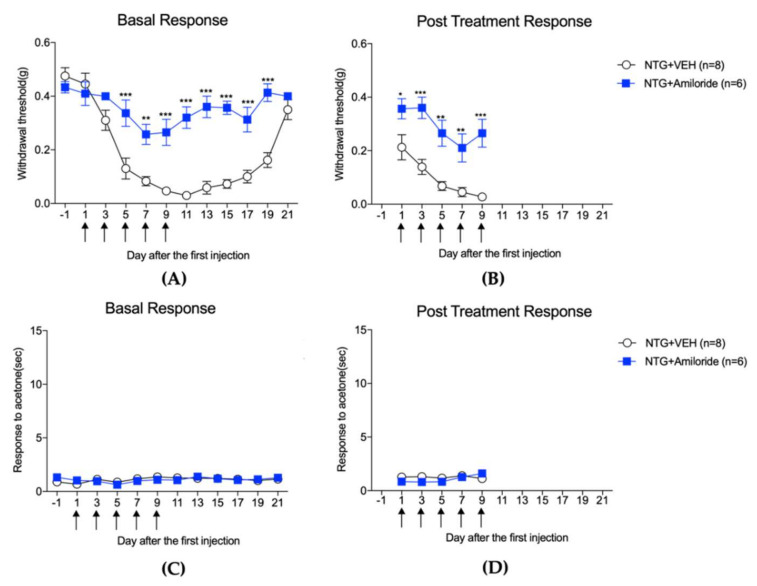
The effect of amiloride (10 mg/kg) treatment on pain responses to mechanical (**A**,**B**) and cold stimulation (**C**,**D**) of hindpaw after repetitive NTG injection. NTG injection decreased the mechanical withdrawal threshold (**A**,**B**), while cold allodynia was not present in either the basal or post-treatment response (**C**,**D**). Amiloride treatment significantly increased the mechanical threshold 2 h after NTG injection (**B**), the post-treatment response; * *p* < 0.05, ** *p* < 0.01, and *** *p* < 0.001 as compared to the NTG + VEH group). In addition, the basal response effect was present 5 days after NTG injection and was maintained even after the treatment with amiloride was complete (**A**), the basal response; ** *p* < 0.01, *** *p* < 0.001 as compared to that in the NTG + VEH group). On the other hand, amiloride did not affect the withdrawal response to acetone in either the basal or post-treatment response (**C**,**D**).

**Figure 2:**
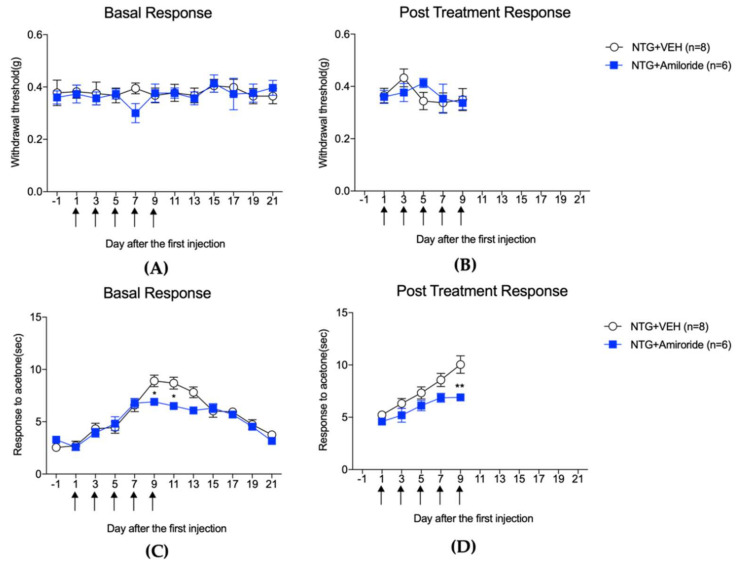
The effect of amiloride (10 mg/kg) treatment on pain responses to mechanical (**A**,**B**) and cold stimulation (**C**,**D**) of facial region after repetitive NTG injection. NTG injection did not affect the mechanical withdrawal threshold (**A**,**B**), while cold allodynia gradually occurred in either the basal or post-treatment response (**C** and **D**). Amiloride treatment did not modify mechanical threshold on the basal response (**A**) or 2 h after NTG injection (**B**). On the other hand, amiloride suppressed the withdrawal response to acetone on the final day of NTG injection ((**C**,**D**), * *p* < 0.05 and ** *p* < 0.01 compared with the NTG + VEH group), and this effect was maintained for 2 days ((**C**), the basal response).

**Figure 3:**
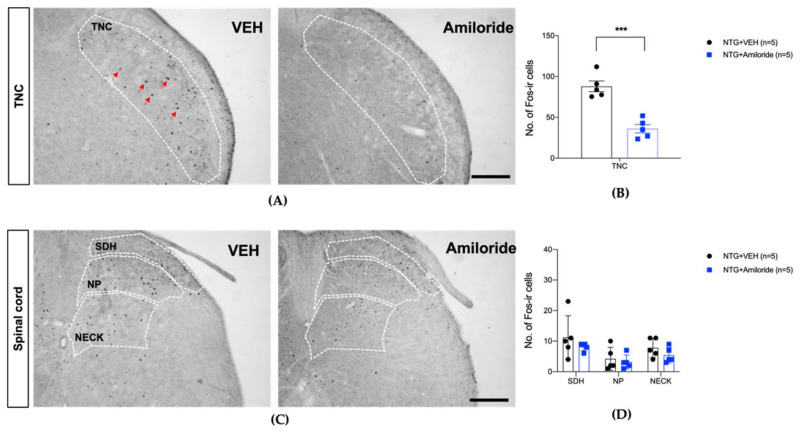
The effect of amiloride (10 mg/kg) on c-Fos expression in the trigeminal nucleus caudalis (TNC, (**A**,**B**)) and lumbar spinal cord dorsal horn (**C**,**D**). The expression of c-Fos protein in the TNC region increased in the NTG-injected animal group (**A**). The amiloride treatment significantly reduced the NTG-induced increase in c-Fos protein expression in the TNC ((**A**,**B**), *** *p* < 0.001 as compared to that in the NTG + VEH group). On the other hand, c-Fos protein expression in the lumbar region of the spinal cord did not increase in the NTG treatment group, and amiloride treatment also produced no significant effect ((**C**,**D**), SDH, superficial dorsal horn; NP, nucleus proprius; NECK, neck of dorsal horn). The red arrows indicate representative c-Fos positive cells. Scale bar = 200 μm.

Figure 1 and Figure 2 depict the mechanical threshold and cold allodynic responses to acetone were evaluated in the hindpaw and whisker pad, respectively, of NTG-injected mice. In addition, the effect of amiloride (10 mg/kg) on c-Fos expression in the TNC (A and B) and lumbar spinal cord dorsal horn (C and D) is shown in Figure 3.

**Figure 4:**
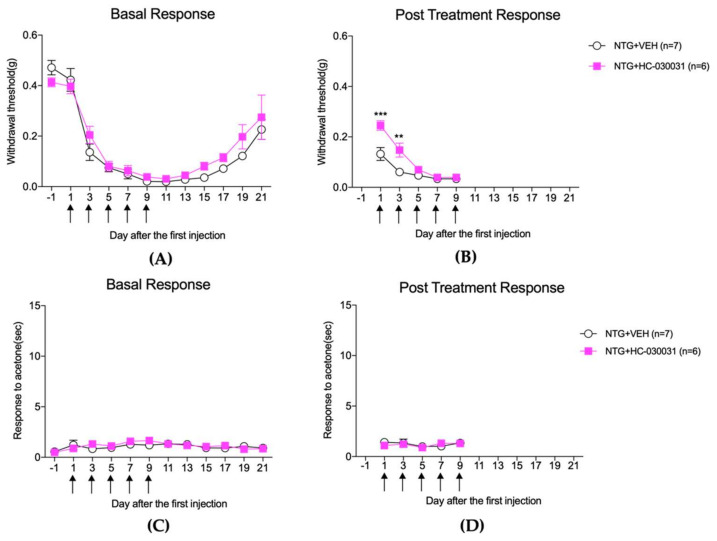
The effect of HC-030031 (100 mg/kg) treatment on pain responses to mechanical (**A**,**B**) and cold stimulation (**C**,**D**) in the hindpaw after repetitive NTG injection. HC-030031 treatment significantly increased the mechanical threshold 2 h after NTG injection on days 1 and 3 ((**B**), the post-treatment response; ** *p* < 0.01 and *** *p* < 0.001 as compared to that in the NTG + VEH group). However, these effects were transient and disappeared in the basal response before each NTG treatment (**A**). Cold allodynia to acetone stimulation did not occur in the hindpaw after NTG injection, and no significant change was observed after HC-030031 treatment (**C**,**D**).

**Figure 5:**
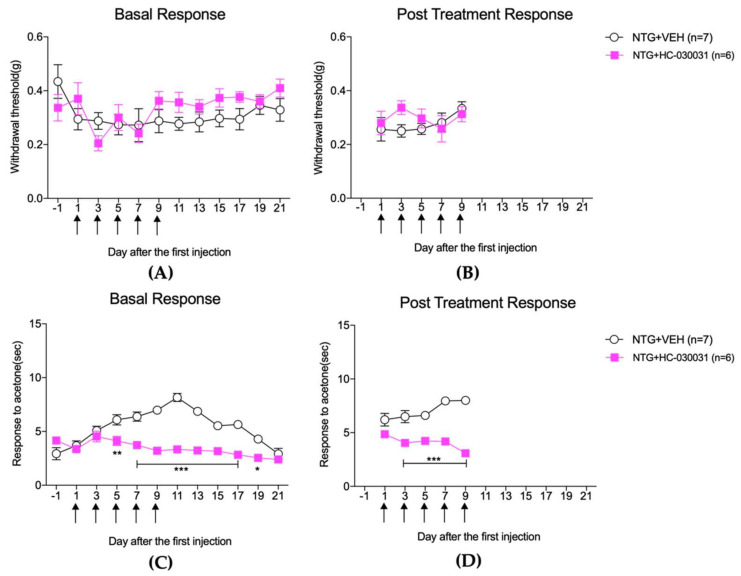
The effect of HC-030031 (100 mg/kg) treatment on pain responses to mechanical (**A**,**B**) and cold stimulation (**C**,**D**) in the facial region of mice after repetitive NTG injection. HC-030031 treatment produced no effect on mechanical threshold on the basal response (**A**) or 2 h after NTG injection (**B**). On the other hand, the basal response to cold allodynia was suppressed from 5 to 17 days after the first injection of NTG ((**C**), the basal response, * *p* < 0.05, ** *p* < 0.01, and *** *p* < 0.001 compared with that in the NTG + VEH group). In addition, cold allodynia caused by acetone stimulation was significantly suppressed 2 h after NTG injection from 3 to 7 days ((**D**), the post-treatment response; *** *p* < 0.001 compared with that in the NTG + VEH group).

**Figure 6:**
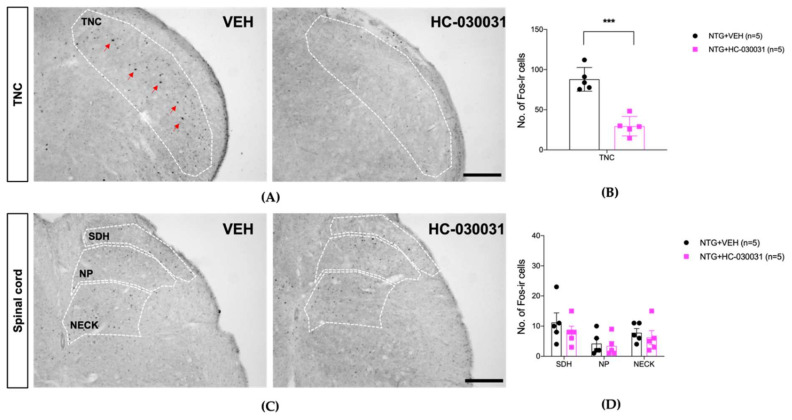
The effect of HC-030031 (100 mg/kg) on the c-Fos expression in the trigeminal nucleus caudalis (TNC, (**A**,**B**)) and lumbar spinal cord dorsal horn (**C**,**D**). The HC-030031 treatment significantly reduced the NTG-induced increase in c-Fos protein expression in the TNC ((**A**,**B**), *** *p* < 0.001 as compared to that in the NTG + VEH group). On the other hand, HC-030031 treatment did not produce a significant suppressive effect on c-Fos expression in the lumbar dorsal horn ((**C**,**D**), SDH, superficial dorsal horn; NP, nucleus proprius; NECK, neck of dorsal horn). The red arrows indicate representative c-Fos positive cells. Scale bar = 200 μm.

Figure 4 and Figure 5 illustrate the effect of HC-030031 on mechanical and cold sensitivity in the hindpaw and facial regions, respectively, of the repeated NTG mouse model, and Figure 6 shows its effect on the c-Fos expression in the TNC and lumbar spinal cord dorsal horn.

## 4. Discussion

Migraine patients have altered sensory stimulus processing in response to lower thresholds of mechanical, thermal, and cold noxious stimuli, resulting in a higher frequency of discomfort and pain than normal individuals [24]. In clinical observations of migraine patients, skin hypersensitivity occurs not only in the head and neck, which are dominated by the trigeminal nerve but also in the distal extremities [25,26]. In our previous study, we developed a mouse migraine model to measure pain hypersensitivity in both the hindpaw and facial regions, which showed that NTG induces hypersensitivity responses to various external stimuli [11]. In addition, these hypersensitivity responses differed by measurement site (hindpaw vs. face). Mechanical allodynia occurred in the hindlimb but not the face, while cold allodynia only occurred in the facial region. These results showed that the development of peripheral hypersensitivity responses in migraine patients can differ depending on the location of the stimulation site and the modality of stimulation. In addition, these results are similar to those of clinical trials, which have reported that the pattern of pain expression in migraine patients may differ depending on the body part being tested [27].

In general, allodynia in the head and neck region is considered to be a kind of referred pain caused by the activation of the trigeminal nervous system. On the other hand, peripheral hyperalgesia in non-cephalic regions is thought to be related to the neuronal activity of the thalamus [25,27]. In this regard, the present study also demonstrated that the mechanism of occurrence of cephalic region and peripheral skin allodynia may be different. We previously confirmed that NTG injection could result in mechanical or cold allodynia in the hind paw and facial region [11]. Thus, whether the systemic injection of NTG increased c-Fos protein expression in both the TNC and spinal dorsal horn was examined. We hypothesized that if mechanical allodynia of the hind paw is directly correlated with the systemic effects of NTG, spinal c-Fos expression can increase along with mechanical allodynia of the hind paw. Mechanical allodynia of the hind paw might also be associated with NTG-induced TNC stimulation, which results in increased c-Fos expression leading to neuronal activation in the central nervous system, including the thalamus. These changes in the higher brain center are likely to cause mechanical hypersensitivity in the non-cephalic region. In Figure 3 and Figure 6, we showed that NTG injection increased the c-Fos expression in the TNC region but not in the lumbar spinal dorsal horn. This indicates that the mechanical allodynia present in the hindpaws may not be related to the central sensitization within the level of the spinal cord. In addition, taken together with several studies, we demonstrate that systemic NTG injection produces migraine-like responses combined with increased neuronal activity in the TNC region. Recently, Verkest et al. showed that the local intra-plantar injection of Mamb-1, a specific inhibitor of the ASIC1a- and ASIC1b-containing channels, completely reversed inflammatory-induced chronic paw mechanical allodynia but not isosorbide dinitrate (ISDN)-induced chronic paw mechanical allodynia [28]. Thus, the systemic extra-cephalic, anti-allodynic effect of i.v. Mamb-1 cannot be attributed to the local inhibition of peripheral sensory ASIC1-containing channels in the paw. In contrast, it is caused by cephalic ISDN-induced reversal of allodynia via the inhibition of ASIC1-containing channels in TG sensory fibers. Peripheral sensitization of trigeminal ganglion (TG) sensory neurons leads to secondary central sensitization of the second-order neurons of the TNC and the upper cervical spinal cord (C1–C2), thereby causing subsequent cephalic allodynia. Meanwhile, extra-cephalic allodynia could reflect a further extension of central sensitization to the upper central pain relays (similar to that in the thalamus), particularly during the settlement of a chronic state [29]. In the current study, mechanical allodynia was observed in the hind paw but not in the facial region. Moreover, it was inhibited with c-Fos expression in the TNC region via the systemic injection of amiloride, a non-selective ASIC blocker. Hence, mechanical allodynia of the hind paw may be caused by the TG-TNC-mediated central mechanism induced by systemic NTG. However, a more in-depth study is needed to elucidate the differential characteristics of pain expression according to body parts reported in migraine patients.

In this study, the treatment of amiloride, a non-selective ASIC blocker, showed a significant inhibitory effect on the development of mechanical allodynia in the hindlimb that was induced by repeated NTG treatment. The short-term effect of amiloride was strong (the post-treatment response), and it also continuously suppressed mechanical allodynia in the hindlimb for more than 10 days after the termination of the drug treatment (the basal response). On the other hand, in the case of cold allodynia in the facial region, no acute effect of amiloride was observed. However, when administered continuously, the cold allodynia caused by NTG injection was relieved on the 9th day of drug administration, and the effect was maintained for 2 days after the drug treatment was finished. These results demonstrate that ASIC channels are deeply involved in the induction and maintenance of mechanical allodynia in the hindlimb and that cold allodynia in the face can be partially modulated by repeated treatment of ASIC channel blockers.

By contrast, other studies have reported that systemic NTG or ISDN injection can induce mechanical allodynia in the periorbital region in rats or mice [28,30]. Verkest et al. showed that the systemic injections of amiloride and mambalgin-1, a specific inhibitor of the ASIC1a- and ASIC1b-containing channels reversed cutaneous mechanical allodynia in the cephalic and extra-cephalic regions [28]. Holton et al. revealed that the ASIC3 blocker APETx2 inhibits neurovascular-evoked and NO-induced sensitization of trigeminal nociceptive responses in rats. In addition, NTG-induced acute and chronic periorbital mechanosensitivities are reversed by APETx2 in mice. Based on these findings, several types of ASICs are involved in migraine-associated mechanical allodynia in both the hind limb and periorbital region. Compared with our previous and current studies, there is a discrepancy in the existence of facial mechanical allodynia, which may be attributed to variations in (1) testing methodology in the facial region (e.g., animals need more time to acclimatize to periorbital stimuli); (2) facial regions tested (periorbital vs. whisker pad); and (3) species (rat vs. mouse), strain (C57BL/6J vs. C57BL/6NTac), and physiological condition (e.g., body weight or age) in rodents. This phenomenon has several causes. However, we believe that our study data are relevant because we have repeatedly confirmed the absence of mechanical allodynia in the facial region of NTG-induced hypersensitivity mice.

A recent review paper suggested that ASIC channels contribute to altered activity in the hypothalamus, cortical spreading depression, and afferent sensory nerve activity in the meninges, and thus ASIC may be a new therapeutic target for migraine patients [31]. Although a direct mechanistic link between ASIC and migraine has not been clearly established, several data, including that given in our present study, have demonstrated the analgesic efficacy of ASIC blockers in preclinical migraine models and clinical trials.

In addition, TRPV1 receptor antagonists are being recently considered for use as a treatment for migraine [14,15]. TRPV1 is known to respond mainly to thermal hyperalgesia, and we also reported that depleting capsaicin-sensitive primary neurons by treatment with resiniferatoxin significantly suppressed only hyperalgesia to heat stimulation among NTG-derived hypersensitivities in the face and hind paw [11]. On the other hand, receptors such as TRPA1 and TRPM8 have been identified as receptors for cold stimulation, and thus, they continue to be applied to research on the development of cold hyperalgesia and allodynia. Demartini et al. confirmed the effect of ADM 12, a TRPA1 antagonist, through a facial formalin pain test in a rat model of NTG-derived migraine induction [18]. In this study, a single administration of NTG significantly amplified the normal pain response caused by facial formalin stimulation, and the ADM 12 drug significantly inhibited the nociceptive response in phase II (late phase), which is a known inflammatory pain response. Moreover, according to a recent report, the isopetasin substance contained in butterbur specifically acts on the TRPA1 channel, leading to an anti-migraine effect in experimental animal models [32]. In particular, it was observed that responses to substance P in the TG nerves could be inhibited by genetic ablation of TRPA1 and treatment with pharmacological antagonists. These results showed that the activity of TRPA1 is important in pain-mediated neurons containing neuropeptides, such as calcitonin gene-related peptide (CGRP) or substance P. Marics et al. also reported that the release of CGRP from the dura mater can amplify the trigeminovascular response, which is closely mediated by TRPA1 [33].

HC-030031, used in this study as a selective TRPA1 antagonist, significantly inhibited the development of facial cold allodynia caused by repeated NTG injection from the third day after treatment (the post-treatment response), and this effect was maintained until the 19th day after the first NTG treatment (the basal response). On the other hand, in the case of mechanical allodynia in the hind limbs of mice, the effect was temporary after HC-030031 treatment on the first and third days after NTG treatment, but the effect did not persist. These results demonstrated that inhibition of TRPA1 through HC-030031 contributes significantly to the suppression of facial cold allodynia but can only temporarily relieve mechanical allodynia of the hind limbs.

In terms of drug concentration, the doses of amiloride and HC-030031 were chosen based on previous studies. Amiloride is systemically injected at a dose of 10 mg/kg to determine its antinociceptive or neuroprotective effect related to pain (migraine) and experimental autoimmune encephalomyelitis in rodent models [28,34]. By contrast, the systemic injection of HC-030031 at a dose of 18–300 mg/kg has been used in several animal models [35,36,37]. Eid et al. reported that HC-030031 at a dose of 100 mg/kg reduced AITC-induced nocifensive behaviors. Moreover, oral HC-030031 at a dose of 100 mg/kg significantly reversed mechanical hypersensitivity in the more chronic models of Complete Freunds Adjuvant-induced inflammatory pain and the spinal nerve ligation model of neuropathic pain. These effects were similar to those of HC-030031 at a dose of 300 mg/kg [35]. In addition, Pereira et al. showed that HC-030031 dose-dependently produced a significant antinociceptive effect against ifosfamide, mustard oil, acetic acid, zymosan, and misoprostol-induced nociception. The maximal effect was observed at a dose of 75 mg/kg [36]. Jain et al. showed that HC-030031 at a dose of 100 mg/kg prevented colitis-associated mechanical hypersensitivity in the abdominal and facial skin areas [37]. Considered together, these results indicate that the systemic injection of HC-030031 at a dose of 100 mg/kg can effectively block TRPA1, which is similar to that in TRPA1 knockout mice [38].

Moreover, TRPA1 is involved in the development of mechanical hypersensitivity in various pain animal models [35,36,37,38]. Marone et al. showed that TRPA1 expressed in trigeminal nociceptors is sensitive to oxidative stress, thereby resulting in delayed and prolonged mechanical allodynia of the periorbital region in NTG-injected mice [38]. Further, they revealed that mechanical allodynia was not observed in TRPA1-deficient mice and was reversed by HC-030031 at a dose of 100 mg/kg. The current study also showed that mechanical allodynia in the hind limbs was transiently relieved with HC-030031 on the first and third days after NTG treatment. However, there was no sustained effect (Figure 4A,B). Based on these results, the pharmacological inhibition of TRPA1 with HC-030031 significantly suppressed the development of mechanical allodynia of the hind paws, but only transiently. Similar to the genetic depleting effect of TRPA1, HC-030031 at a dose of 100 mg/kg can effectively block TRPA1 [38]. Thus, the activation of TRPA1 is closely correlated with the development of cold allodynia in the facial region. Meanwhile, the induction of mechanical allodynia in the hind limb may be temporarily affected by HC-030031 treatment.

Notably, both the ASIC channel blocker treatment and the TRPA1 receptor antagonist treatment showed similar levels of inhibition of c-Fos protein expression in the TNC region. Although the effects of the two drugs are markedly different depending on the stimulation site and type of stimulation, the level of c-Fos protein expression is similar. Therefore, the change in c-Fos expression in the TNC mediates the pain response to all stimuli. More importantly, the effect of amiloride did not affect c-Fos expression in the lumbar spinal cord, although it suppressed mechanical allodynia in the hind limb. This finding indicates that mechanical allodynia in the hind limb caused by NTG injection is also closely related to the activity of the TNC neurons, and it indirectly shows that hindpaw mechanical allodynia may be one of the NTG-induced migraine-like symptoms. However, it seems that the relationship between the regulation of c-Fos protein expression in the TNC region and the regulation of migraine-related pain patterns needs to be further elucidated through additional studies.

## 5. Conclusions

This study compared and analyzed the pattern of pain in the face and hind limbs using a migraine mouse model induced by repeated NTG injection. The activation of ASICs contributed to the development of mechanical allodynia of the hindlimb, while the activation of the TRPA1 receptor was mainly associated with the induction of facial cold allodynia. In addition, both ASICs and TRPA1 mediated the modulation of c-Fos expression in the TNC but not in the lumbar spinal cord. These findings demonstrate that facial cold allodynia and mechanical allodynia of the hindlimb are induced by repetitive NTG injection and are differentially mediated by the activation of TRPA1 and ASICs, respectively.

## Data Availability

All the data in support of the findings presented are included and can be available by the corresponding author with any request.
